# Redox signalling by protein kinase G Iα in cardiovascular physiology and pathology

**DOI:** 10.1186/2050-6511-14-S1-O19

**Published:** 2013-08-29

**Authors:** Philip Eaton

**Affiliations:** 1 Cardiovascular Division, King’s College London, London, SE1 7EH, UK

## Background

Proteomic studies allowed us to identify PKA RIα and then PKG Iα as kinases that form interprotein disulfides in response to oxidants such as hydrogen peroxide (H_2_O_2_). This oxidation event directly activated PKG Iα independently of the classical NO-cGMP pathway to cause vasodilation. Subsequently we generated a Cys42Ser PKG Iα ‘redox-dead’ knock-in (KI) mouse. PKG Iα in these KI mice cannot be oxidant-activated as it lacks the thiol redox sensor. Consequently KI blood vessels do not relax fully to oxidants or endothelium derived hyperpolarising factor (EDHF) stimuli - resulting in hypertension in vivo compared to wild-type (WT) littermates. This provided robust evidence PKG Iα oxidation is a significant mechanism of lowering BP in vivo. Additional studies showed cGMP binding to PKG induces a state that is resistant to disulfide formation. Thus interventions that lower cGMP stimulate PKG oxidation. Consequently, PKG oxidation occurs to a lesser extent in aortas than in mesenteries, as conduit vessels have higher levels of NO. Conduit vessels also express more peroxiredoxin and thioredoxin than resistance vessels, perhaps allowing oxidants such as H_2_O_2_ accumulate at higher levels in the latter. Together this helps explain why resistance vessels, principal regulators of blood pressure, are highly sensitive to PKG Iα oxidation and consequently oxidant-induced vasodilation compared to conduits.

## Results

We hypothesized that nitroglycerin-induced vasodilation may involve disulfide activation of PKG Iα. We reasoned this as nitroglycerin is not simply an NO-donor and is bioactivated to a molecular form with oxidant properties. Indeed, we found that nitroglycerin induced PKG Iα oxidation in cells and tissues, and that isolated mesenteries from KI mice were significantly resistant to nitroglycerin-induced vasodilation compared to WT. Consistent with this when nitroglycerin was administered by osmotic mini-pump to WT and KI mice in vivo, the blood pressure-lowering was markedly abrogated in the latter. We also hypothesized PKG Iα oxidation perhaps mediated sepsis-induced hypotension leading to organ under-perfusion and injury. This was considered a rational possibility as sepsis is a time of nitro-oxidative stress and a cardinal feature of the disease is hypotension. KI mice were resistant to hypotension induced by two common models of murine sepsis (namely lipopolysaccharide or ceacal ligation and perforation) compared to wild-type controls. Consistent with this, the KI (presumably due to preserved blood pressure during sepsis) showed less end-organ damage and dysfunction compared to WT.

## Conclusion

Overall we conclude that PKG Iα oxidation is a major mechanism that controls blood pressure in health, but during sepsis this mechanism can be over-stimulated to induce hypotension, leading to tissue injury which compromises well-being and survival.

